# Hearing Loss in HIV-Infected Children in Lilongwe, Malawi

**DOI:** 10.1371/journal.pone.0161421

**Published:** 2016-08-23

**Authors:** Susan Hrapcak, Hannah Kuper, Peter Bartlett, Akash Devendra, Atupele Makawa, Maria Kim, Peter Kazembe, Saeed Ahmed

**Affiliations:** 1 Baylor College of Medicine Abbott Fund Children’s Clinical Centre of Excellence Malawi, Lilongwe, Malawi; 2 Department of Pediatrics, Baylor College of Medicine, Houston, Texas, United States of America; 3 Baylor International Pediatric AIDS Initiative, Texas Children’s Hospital, Houston, Texas, United States of America; 4 International Centre for Evidence in Disability, London School of Hygiene & Tropical Medicine, London, United Kingdom; 5 African Bible College (ABC) Hearing Clinic and Training Centre, Lilongwe, Malawi; University of South Florida, UNITED STATES

## Abstract

**Introduction:**

With improved access to antiretroviral therapy (ART), HIV infection is becoming a chronic illness. Preliminary data suggest that HIV-infected children have a higher risk of disabilities, including hearing impairment, although data are sparse. This study aimed to estimate the prevalence and types of hearing loss in HIV-infected children in Lilongwe, Malawi.

**Methods:**

This was a cross-sectional survey of 380 HIV-infected children aged 4–14 years attending ART clinic in Lilongwe between December 2013-March 2014. Data was collected through pediatric quality of life and sociodemographic questionnaires, electronic medical record review, and detailed audiologic testing. Hearing loss was defined as >20 decibels hearing level (dBHL) in either ear. Predictors of hearing loss were explored by regression analysis generating age- and sex-adjusted odds ratios. Children with significant hearing loss were fitted with hearing aids.

**Results:**

Of 380 patients, 24% had hearing loss: 82% conductive, 14% sensorineural, and 4% mixed. Twenty-one patients (23% of those with hearing loss) were referred for hearing aid fitting. There was a higher prevalence of hearing loss in children with history of frequent ear infections (OR 7.4, 4.2–13.0) and ear drainage (OR 6.4, 3.6–11.6). Hearing loss was linked to history of WHO Stage 3 (OR 2.4, 1.2–4.5) or Stage 4 (OR 6.4, 2.7–15.2) and history of malnutrition (OR 2.1, 1.3–3.5), but not to duration of ART or CD4. Only 40% of caregivers accurately perceived their child’s hearing loss. Children with hearing impairment were less likely to attend school and had poorer emotional (p = 0.02) and school functioning (p = 0.04).

**Conclusions:**

There is an urgent need for improved screening tools, identification and treatment of hearing problems in HIV-infected children, as hearing loss was common in this group and affected school functioning and quality of life. Clear strategies were identified for prevention and treatment, since most hearing loss was conductive in nature, likely due to frequent ear infections, and many children with hearing loss qualified for hearing aids. Screening strategies need to be developed and tested since caregivers were not reliable at identifying hearing loss, and often mis-identified children with normal hearing as having hearing loss. Children with frequent ear infections, ear drainage, TB, severe HIV disease, or low BMI should receive more frequent ear assessments and hearing evaluations.

## Introduction

HIV currently affects an estimated 35 million people worldwide. Over the past decade, quality HIV care and access to ART has become much more widely available throughout Africa. The expansion in access to ART globally has reduced HIV-related morbidity and mortality [1,2] and contributed to an increase in life expectancy, including in low- income countries; in many instances, it is now comparable to the general population [3]. As a consequence, HIV infection is becoming a chronic illness [4,5]. As more HIV-infected patients are surviving, the magnitude of a variety of disabilities has become more evident. These disabilities can result from the HIV virus itself, opportunistic infections, or from medication side effects [4]. These disabilities can further disadvantage children living with HIV, although the impact on their lives has rarely been investigated.

One million of these HIV- infected patients live in Malawi, and 150,000 are children less than 15 years of age [6]. Malawi has achieved a remarkably successful scale-up of HIV care and treatment services. Whilst pediatric services initially lagged behind, in recent years there have been great strides in expanding coverage for all children living with HIV. There are currently around 42,525 children less than 15 years of age alive and on ART, representing nearly 9% of all people on ART in Malawi [7].

To date, there are few studies looking at hearing loss in HIV-infected children, and many have been small in size. All have found a high prevalence of hearing loss in HIV-infected children, ranging between 6.4–84.8% [8–17], which is much higher when compared to general population studies in children in these countries (4% in US, 6.9% in Peru) [8,10] or to uninfected controls (8.3% in South Africa) [13]. Hearing loss in HIV can arise from a variety of causes, including recurrent otitis media, opportunistic infections such as cytomegalovirus (CMV), tuberculosis (TB) or cryptococcus, syphilis, bacterial meningitis, or from side effects of medications such as gentamicin or streptomycin [18]. Some research has even suggested that some of the antiretroviral medications, such as certain nucleoside reverse transcriptase inhibitors (NRTIs), may potentially lead to sensorineural hearing loss [19], although additional research has suggested that antiretroviral medications do not result in hearing loss [20]. The nature of the relationship between HIV and hearing loss, is therefore not yet clear, nor have strategies for screening, prevention and treatment been outlined.

A recent study at the Baylor College of Medicine Clinical Centre of Excellence (BCOE) in Lilongwe, Malawi reported higher prevalence of a range of disabilities in children living with HIV when compared to their HIV-uninfected siblings [21]. Overall, 33% of HIV-infected children aged 2–9 years screened positive for a disability versus only 7% of their HIV-uninfected siblings; of the disabilities reported, 35% were hearing-related. Of the total patients participating in the study, caregivers reported hearing difficulties in 12% of HIV-infected children, compared with only 2% of their uninfected siblings. This current study was developed in response to the high reports of hearing difficulties from the prior study, with the primary aim to use audiologic assessments to assess the prevalence and types of hearing loss in HIV-infected children attending the BCOE in Lilongwe, Malawi. Secondary aims included characterizing clinical and sociodemographic factors associated with hearing loss in HIV-infected children, in order to identify strategies for prevention and treatment, as well as assessment of the impact of hearing loss on quality of life.

## Materials and Methods

### Ethical considerations

This study was reviewed and approved by both the Malawi National Health Sciences Research Committee (NHSRC Protocol #1200) and the Baylor College of Medicine Institutional Review Board (BCM Protocol H-33667). Full written informed consent was obtained from adult caregivers, and assent was obtained from children 10 years and older unless they did not have adequate cognitive ability to understand the study due to disability. Children who did not have cognitive ability for assent were included in the study, as long as their caregiver provided informed consent and a study clinician documented inability of the child to provide assent due to cognitive delay. Participation in the study was entirely voluntary and confidential, and participants were free to withdraw at any time without any consequences.

### Study Design and Setting

This was a cross-sectional study conducted from December 2013 to March 2014.

### Study Setting and Participants

Participants were HIV-infected children 4 to 14 years of age attending the Baylor College of Medicine Children’s Clinical Center of Excellence (BCOE) in Lilongwe, Malawi. The BCOE in Lilongwe serves as a tertiary referral center for pediatric HIV care and treatment, and has approximately 3500 patients actively enrolled. This represents the largest cohort of pediatric HIV-infected children in Malawi. While data are not available for all children participating in the study, the vast majority of children attending care at BCOE are perinatally-infected. Participants were selected from a convenience sample of HIV-infected children aged 4 to 14 years who were attending clinic at the BCOE and who were accompanied by their primary caregiver. Caregiver report of hearing loss was not a criterion for enrollment. After informed consent was completed, a research assistant completed two questionnaires with the caregiver and/or child. Of the 380 participants, 93 children completed questionnaires. The children were then transported to African Bible College (ABC) Hearing Clinic and Training Centre, where trained audiology staff performed extensive audiologic testing. ABC is a 5-minute drive from the BCOE. On occasions when large numbers of older children were enrolled, a portable testing van containing two soundproof booths was parked at BCOE and testing was done onsite. Fifty-six children were tested using the audiotrailer. Younger children and children unable to be assessed using age-appropriate subjective methods required the larger booths at ABC for play audiometry, visual reinforcement audiometry (VRA), and other objective diagnostic assessment equipment. Both the audiotrailer and the permanent audiobooths at ABC were custom built in South Africa by the company Audiometry Specialists, using the same materials for the paneling. The audiotrailer was constructed to meet industrial hearing conservation program standards for mobile testing units. The ambient noise levels of the audiotrailer are assessed annually by an independent accredited calibrator from Johannesburg, using South African Bureau of Standards (SABS) 0182/ 1998 maximum allowable noise measurements in dB of sound pressure level (dBSPL). All relevant octave and interoctave frequency measures from ambient noise from 125 Hz to 8KHz were under the maximum allowable limits. The diagnostic audiometers in the audiotrailer were calibrated by the same technician who calibrates the audiometers in the hearing clinic booths and measures the ambient noise levels of the audiotrailer. Testing location was not included in statistical analysis.

### Data Collection and Tools

Sociodemographic information was collected on age, gender, caregiver education, caregiver HIV status, household size, household location, family income, source of water, asset ownership, and food security. Information was also collected on school attendance and performance, history of medical illnesses, and signs of hearing impairment. If a caregiver reported that they believed their child had hearing impairment, an additional set of questions was asked concerning the nature of the disability (severity, prior referrals, rehabilitation services, assistive devices, and perceived barriers and challenges). The following HIV-related data were extracted from the electronic medical records at BCOE on the date of the audiology testing: timing and duration of ART, CD4+ cell count, WHO status at enrollment, current WHO status, history of TB, nutritional status, history of streptomycin exposure, documented history of hearing or ear problems. All children on ART were on combination antiretroviral therapy (cART) with three antiretroviral medications. Regimens were demarcated as non-nucleoside reverse transcriptase inhibitor (NNRTI) or protease inhibitor (PI) based regimens. History of malnutrition was determined by documented weight for height less than the 85^th^ percentile as per the Ministry of Health guidelines at the time of the study [22].

Caregivers completed the Caregiver version of the Pediatric Quality of Life Inventory (PedsQL^™^), which has been translated into Chichewa and validated in Malawi [21]. The questionnaire contains items on physical functioning (8 questions), emotional functioning (5 questions), social functioning (5 questions), and school functioning (3–5 questions). Each question was rated on a five-point scale from 0 (never a problem) to 4 (almost always a problem). Separate questionnaires were used for children aged 4, 5–7, 8–12, and 13–14. Children aged 10 and above completed the Child version of the PedsQL^™^ within the appropriate age category if they had assented and were cognitively able to understand the questionnaire.

Trained audiology staff at ABC Hearing Clinic and Training Centre completed hearing analysis on all children, including the following: otoscopy, tympanometry, transient evoked otoacoustic emissions (TEOAE), and audiometry. The AccuScreen Pro was used for TEOAE testing and uses a non-linear click sequence stimulus with click rate of approximately 60 Hz and sampling rate of 16 kHz. The pass/refer criteria were evaluated according to AccuScreen binomial statistics. The criterion level used by the AccuScreen Pro signal detection algorithm for the presence of a time-locked signal is 99.7%. All audiometric assessment included in the study was age-appropriate and performed under headphones. Pure-tone audiometry using a button or hand-raise response was the preferred method. However, for those children aged 4–6 years, a play task was used to elicit the child’s response. If any cognitive impairment impacted pure-tone or play testing, this was noted by the tester as an issue in preventing a reliable assessment of hearing, and an alternative method was conducted. Eight children were unable to complete pure-tone or play audiometry due to developmental delay; three of these children successfully completed testing using VRA (also known as puppet in window testing) under headphones, and four children successfully completed VRA under freefield. One child required auditory steady state testing under sedation due to severe neurocognitive impairment, with stimulus presented through insert earphones. Audiometry testing was performed using an Amplivox 240 Portable Diagnostic Audiometer at the following frequencies: 500, 1000, 2000, 3000, 4000, 8000 Hz. Bone conduction frequencies were tested at 500 Hz, 1000 Hz, 2000 Hz and 4000 Hz. Pure tone average was used at four frequencies (500, 1000, 2000, and 4000 Hz) to determine the average hearing threshold level. Hearing loss was defined as greater than 20 decibels hearing level (dBHL) in either ear. Categories of hearing loss were defined as follows: normal (up to 20), mild (21–40), moderate (41–65), severe (66–90), and profound (≥91). The type of hearing loss was determined based on the relationship of the four air and bone conduction thresholds. If the bone conduction level was within 10dBHL of the air conduction level, the hearing loss was considered to be sensorineural. If the bone conduction level was within the normal range of hearing (≤20dBHL), then the hearing loss was conductive. If the bone conduction level was outside the normal range, but still better than the air-conduction threshold by more than 10dBHL, then the hearing loss was considered to be mixed. Also, if the hearing loss was clearly conductive at some frequencies and sensorineural at other frequencies, it was considered mixed, based on the definition “a mixed hearing loss is the combination of a sensorineural loss and a conductive loss in the same ear” [23].

Children who qualified were fitted with hearing aids. Qualification for hearing aids was determined by an audiologist and was based upon a combination of clinical and social criteria. A bone conduction hearing aid was considered for children with a chronic and recent history of discharging ears who had a mild to severe air conduction audiometric threshold average (30–85 dBHL) in the better ear, and a normal or mild bone conduction audiometric threshold average (≤35 dBHL). An air conduction hearing aid (behind the ear type), was considered for children where their ear did not have ear discharge issues or other contraindications, but had a mild to profound degree of hearing loss (average air conduction of ≥30 to ≤105 dBHL) which was sensorineural, conductive, or mixed in nature. Hearing aid fitting was then discussed with the child and guardian to ensure there was a recognized functional hearing loss requiring rehabilitation and that the device would be appropriately used and cared for.

### Data Analysis

Age- and sex-adjusted odds ratios were generated through logistic regression to assess the relationship between the presence of hearing loss and HIV and sociodemographic characteristics. For all analyses, the case definition was hearing loss greater than 20 dBHL in the worse ear. The PedsQL^™^ were reverse-scored and linearly transformed to a 0–100 scale (0 = 100, 1 = 75, 2 = 25, 3 = 25, 4 = 0) with higher scores indicating better quality of life. Scale scores were computed as the sum of the items divided by the number of items answered. If more than half the items were missing data, then the scale score was not calculated. Age-sex-adjusted logistic regression analyses were then completed to compare those with hearing loss versus no hearing loss.

Sensitivity of TEOAE as a screener for hearing loss was calculated by measuring the proportion of people with hearing loss (as defined by audiometry) who did not pass the TEOAE test. Specificity of TEOAE as a screener was calculated by measuring the proportion of people without hearing loss (as defined by audiometry) who passed the TEOAE test.

## Results

### Prevalence of Hearing Loss

Of the 380 children aged 4 to 14 years recruited and tested, 90 (24%) patients had hearing loss in either ear (mild 15%, moderate 5%, severe 2%, and profound 2%). Overall, 36 (9%) had an average hearing threshold of >20 dBHL in the better ear. Of the children with hearing loss in either ear, 82% of the hearing loss was conductive, 14% was sensorineural, and 4% was mixed. Degrees of hearing loss by type are included in Table 1. For the four children tested under freefield, the ear-specific data included in the analyses was determined using results from the full battery of tests as follows: one child had normal otoscopy, tympanometry, OAEs and VRA with no reported history of hearing loss, so had presumed normal hearing for both ears. Another child had all results consistent with bilateral middle ear pathology, with 40–60 dBHL so a moderate conductive hearing loss was the most likely diagnosis for both ears. A third child had mild hearing loss in freefield, likely to be the right ear, which had pass for OAE despite a type B tympanogram indicative of middle ear effusion. The left ear was assumed to be a moderate conductive loss due to a heavily discharging ear. The fourth child was later able to complete VRA under headphones, and the results of this test were used in the analysis.

**Table 1:** Degrees and Types of Hearing Loss in ears of HIV-infected children in Lilongwe, Malawi.

Degree of Hearing Loss (dBHL)	Sensorineural	Conductive	Mixed
**Mild (21–40)**	6 (35%)	77 (75%)	1 (17%)
**Moderate (41–65)**	1 (6%)	24 (23%)	1 (17%)
**Severe (66–90)**	3 (18%)	2 (2%)	4 (67%)
**Profound (≥91)**	7 (41%)	0 (0%)	0 (0%)
Total*	17	103	6

*Data regarding type of hearing loss is missing for one ear

There is weak evidence that the sensorineural hearing loss is associated with more profound levels of hearing loss, but it is difficult to make a clear statement given the small numbers. Among children with hearing loss, 21 (23%) were referred for hearing aid fitting, 63% were not recommended for hearing aid, and 14% were recommended to be reviewed at a later date. Most children were fitted with bilateral hearing aids; children fitted with unilateral hearing aids had either a very mild high frequency hearing loss in the non-fitted ear or an unaidable profound hearing loss in the non-fitted ear.

### Hearing characteristics (Table 2)

**Table 2:** Relationship with hearing characteristics to hearing loss of the ears in children infected with HIV in Lilongwe, Malawi.

		Ears with hearing loss (n = 127)	Ears without hearing loss (n = 633)	p-value
Tympanometry	Normal	19 (16%)	499 (79%)	<0.0001
	Abnormal	98 (84%)	133 (21%)	
Transient evoked otoacoustic emissions (TEOAE)	Pass	25 (21%)	598 (95%)	<0.0001
	Refer	94 (79%)	31 (5%)	
Otoscopy	Wax	45 (35%)	149 (24%)	0.005
	Discharge	31 (24%)	10 (2%)	<0.001
	Perforated	36 (28%)	12 (2%)	<0.001
	Fungi	6 (5%)	4 (1%)	0.0002
	Red/bulging ear drum	16 (13%)	6 (1%)	<0.001
	Ear drum abnormal	96 (76%)	142 (24%)	<0.001
	Ear drum not seen	23 (18%)	59 (9%)	0.004

Most of the ears with conductive hearing loss had abnormal physical findings. For the 26 ears with moderate or severe conductive hearing loss, 62% had perforated eardrums, 8% had an acute otitis media with intact eardrum, 12% had a fungal infection, 38% had discharge, and 20% had more than one abnormal finding. A higher percentage of patients with hearing loss had discharge in the external ear canal and perforated eardrum at the time of testing than those patients without hearing loss. Other otoscopy findings such as ear canal inflammation, foreign body, or dullness of eardrum were not related to hearing loss. All patients with otitis media, otitis externa, fungal infections, effusions, or other medical issues were treated and re-evaluated at a later date. Patients with discharge and perforation were also evaluated at a later date after the physical exam findings had resolved for repeat testing. Presence of wax was defined if it was obstructing or occluding the ear canal. The eardrum was more likely to be abnormal in those patients with hearing loss. Only 16% of patient’s ears with hearing loss had normal tympanometry. The majority with abnormal tympanometry had no observable middle ear mobility for compliance with normal canal volume (Bmid), indicating a middle ear effusion. In comparison, 79% of ears in patients without hearing loss had type A tympanometry, indicating normal mobility and pressure. A higher percentage of ears in patients with hearing loss did not pass the transient evoked otoacoustic emissions test (TEOAE) when compared with ears without hearing loss. If OAE was used as the screening test for hearing loss, this would have 79% sensitivity and 95% specificity.

### Sociodemographic Factors (Table 3)

**Table 3:** Sociodemographic Factors of HIV-infected Children in Lilongwe, Malawi in Relation to Hearing Loss.

		Hearing loss (n = 90)	No hearing loss (n = 290)	Age-sex-adjusted OR (95% confidence interval)
Age	4	13 (15%)	34 (12%)	Baseline
	5–7	36 (41%)	124 (43%)	1.3 (0.6–2.8)
	8–12	25 (28%)	83 (29%)	1.3 (0.6–2.8)
	13–14	14 (16%)	46 (16%)	1.3 (0.5–3.0)
Gender	Male	44 (49%)	145 (50%)	Baseline
	Female	46 (51%)	145 (50%)	1.0 (0.6–1.5)
Parental Status	One or both parents dead	32 (36%)	86 (30%)	1.4 (0.8–2.4)
	Both parents alive	58 (64%)	204 (70%)	Baseline
Other disabilities	Yes	17 (19%)	31 (11%)	1.8 (1.0–3.5)
	No	73 (81%)	259 (89%)	Baseline
**Socioeconomic Status**				
Asset Score	Low	53 (59%)	144 (50%)	Baseline
	Medium	28 (31%)	78 (27%)	3.1 (1.4–7.0)
	High	9 (10%)	68 (23%)	3.1 (1.3–7.3)
Education of father	None/Primary	35 (44%)	84 (30%)	1.5 (0.9–2.5)
	Secondary+	45 (56%)	193 (70%)	Baseline
Education of Mother	None/Primary	45 (51%)	132 (46%)	1.2 (0.7–1.9)
	Secondary+	43 (49%)	155 (54%)	Baseline
Family income	Low	29 (43%)	109 (46%)	Baseline
	Medium	24 (36%)	73 (31%)	1.3 (0.7–2.4)
	High	14 (21%)	56 (23%)	0.9 (0.4–1.8)
Location	Rural	34 (38%)	123 (42%)	Baseline
	Urban	42 (47%)	126 (44%)	1.2 (0.7–2.1)
	Peri-urban	14 (15%)	41 (14%)	1.2 (0.6–2.5)
Food security	High	18 (20%)	67 (23%)	Baseline
	Medium	38 (42%)	115 (40%)	1.3 (0.7–2.5)
	Low	34 (38%)	108 (37%)	1.2 (0.6–2.3)

Mean age of all participants was 8.6 ± 3.2 years). Ages were similar in those with hearing loss (mean age 8.5 ± 3.3 years) and those without hearing loss (mean age = 8.6 years ± 3.2 years). There was no difference in age or gender comparing those with and without hearing loss. Children with hearing loss tended to be more likely to report experiencing other disabilities. Orphan status and current caregiver were not related to hearing loss. Children from families with higher socioeconomic assets were less likely to have hearing loss than those from lower socioeconomic status. Family income, location (urban vs. rural), food security, and parental levels of education were not related to hearing loss.

### Caregiver perception of hearing loss (Table 4)

**Table 4:** Caregiver Perception of Hearing Loss in HIV-Infected Children in Lilongwe, Malawi.

		Hearing loss (n = 90)	No hearing loss (n = 290)	Age-sex adjusted OR (95% confidence interval)
Perception of hearing loss	Yes	36 (40%)	31 (11%)	5.9 (3.3–10.6)
	No	54 (60%)	259 (89%)	Baseline
If yes, discussed with clinician	Yes	24 (67%)	10 (32%)	4.6 (1.6–13.6)
	No	12 (33%)	21 (68%)	Baseline
Need to repeat for child to understand	Yes	37 (41%)	68 (23%)	5.0 (3.0–8.4)
	No	53 (59%)	222 (77%)	Baseline
Difficulty following instructions	Yes	27 (30%)	48 (17%)	2.2 (1.3–3.9)
	No	63 (70%)	242 (83%)	Baseline
Child speaks clearly	Yes	68 (76%)	261 (90%)	Baseline
	No	22 (24%)	29 (10%)	2.9 (1.6–5.5)
Family history of hearing loss	Yes	15 (17%)	28 (10%)	1.8 (0.9–3.6)
	No	75 (83%)	262 (90%)	Baseline
Sought treatment for hearing loss	Yes	16 (18%)	9 (3%)	7.5 (3.1–18.1)
	No	74 (82%)	281 (97%)	Baseline
Hearing problem noted in EMR	Yes	55 (61%)	111 (38%)	2.7 (1.6–4.4)
	No	35 (39%)	179 (62%)	Baseline

Only 40% of caregivers of children with hearing loss accurately perceived the hearing loss, though hearing impairment was related to caregiver perceived hearing loss. Of those with hearing loss perceived by the caregiver, 67% had previously discussed the concern with a clinician. Those with hearing loss were more likely to have had a hearing or ear problem noted in the electronic medical record (EMR) and were more likely to have previously sought treatment. Positive answers to caregiver screening questions were related to hearing loss, including needing to repeat things to the child, the child having difficulty following instructions, and the child not speaking clearly. Family history of hearing loss was not related to presence of hearing loss.

Among the children with perceived hearing loss, caregivers attributed this to unknown causes (60%), illness (30%) and rarely to HIV (10%). Caregivers noted hearing loss at an average of 6.0 years (SD = 3.0 years). Among the children with hearing loss by audiometric testing, only 7 (19%) had received a previous hearing test, and 3 of these children had already been fitted with hearing aids prior to the study. Services sought for hearing loss included hospital (25%), specialist clinic (22%), private doctor (6%) and other services (3%); the remaining children had not sought any services.

### Hearing Loss in relation to other health conditions (Table 5)

**Table 5:** Hearing Loss in Relation to Other Health Conditions in HIV-infected Children in Lilongwe, Malawi.

		Hearing loss (n = 90)	No hearing loss (n = 290)	Age-sex adjusted OR (95% confidence interval)
Place of birth	Hospital	61 (68%)	170 (59%)	1.1 (0.5–2.7)
	Health clinic	21 (23%)	93 (33%)	0.7 (0.3–1.8)
	Home	8 (9%)	24 (8%)	Baseline
History of overnight admission	Yes	74 (82%)	228 (79%)	1.3 (0.7–2.4)
	No	16 (18%)	62 (21%)	Baseline
Recent outpatient treatment for illness (<2 months)	Yes	13 (14%)	29 (10%)	1.5 (0.7–3.1)
	No	77 (86%)	261 (90%)	Baseline
Meningitis	Yes	6 (7%)	17 (6%)	1.2 (0.5–3.2)
	No	84 (93%)	273 (94%)	Baseline
Frequent ear infections	Yes	42 (47%)	31 (11%)	7.4 (4.2–13.0)
	No	48 (53%)	259 (89%)	Baseline
Ear drainage	Yes	35 (39%)	27 (9%)	6.4 (3.6–11.6)
	No	55 (61%)	263 (91%)	Baseline
TB treatment	Yes	43 (48%)	105 (36%)	1.7 (1.0–2.7)
	No	47 (52%)	185 (64%)	Baseline
BMI (body mass index)	BMI<16	54 (60%)	185 (64%)	0.8 (0.5–1.4)
	BMI> = 16	36 (40%)	105 (36%)	Baseline

There was a higher prevalence of hearing loss in children with history of frequent ear infections and ear drainage. History of TB treatment tended to be more common among children with hearing loss; only 4 children with hearing loss had been exposed to the ototoxic medication streptomycin as part of their TB treatment. Place of birth, history of overnight admissions, recent outpatient treatment for an illness, history of meningitis, and current body mass index (BMI) were not related to hearing loss.

### Hearing Loss in relation to HIV characteristics (Table 6)

**Table 6:** Hearing Loss in relation to HIV characteristics among children in Lilongwe, Malawi.

		Hearing loss (n = 90)	No hearing loss (n = 290)	Age-sex adjusted OR (95% confidence interval)
On ART	Yes	88 (98%)	268 (92%)	3.8 (0.9–16.4)
	No	2 (2%)	22 (8%)	Baseline
If yes, duration of ART		4.6 (2.2)	4.7 (2.1)	P = 0.67
If yes, age at ART initiation		4.0 (3.2)	3.9 (3.5)	P = 0.72
WHO stage at enrolment	1	15 (17%)	98 (34%)	Baseline
	2	13 (14%)	44 (15%)	2.1 (0.9–4.8)
	3	44 (49%)	129 (44%)	2.4 (1.2–4.5)
	4	18 (20%)	19 (7%)	6.4 (2.7–15.2)
Current WHO stage	1	84 (93%)	281 (97%)	Baseline
	2+	6 (7%)	9 (3%)	2.2 (0.7–6.4)
Nadir CD4		476 (373)	482 (355)	P = 0.57
Nadir CD4<200	Yes	19 (22%)	61 (21%)	1.1 (0.6–2.0)
	No	67 (78%)	223 (79%)	Baseline
Current CD4		935 (510)	935 (499)	P = 0.79
Age entering into care		3.9 (3.4)	3.6 (3.4)	P = 0.19
History of malnutrition recorded in EMR	Yes	48 (53%)	101 (35%)	2.1 (1.3–3.5)
	No	42 (47%)	189 (65%)	Baseline

Almost all children with hearing loss (98%) were on ART, and average duration was 4.6 years. There was no significant difference between the age of entry into care, the age of ART initiation, or duration of ART in children with and without hearing loss. Children with hearing loss were more likely to have been WHO Stage 3 or Stage 4 at enrollment in clinic, rather than Stage 1. Current WHO Stage, and current or nadir CD4 <200 were not related to hearing impairment. For those with any degree of malnutrition recorded in the EMR, there was a higher association with hearing loss. Various ART regimens were not significantly different between children with and without hearing loss.

### Impact of hearing loss on lives of HIV-infected children (Table 7)

**Table 7:** Impact of hearing loss on the lives of children with HIV in Lilongwe, Malawi (age >5).

		Hearing loss (n = 90)	No hearing loss (n = 290)	Age-sex adjusted OR (95% confidence interval)
Attending school	Never	10 (13%)	16 (6%)	2.5 (1.0–6.0)
	Sometimes	8 (11%)	25 (10%)	1.2 (0.5–2.8)
	Always	57 (76%)	212 (84%)	Baseline
If yes, school year correct for age	No	29 (45%)	97 (41%)	Baseline
	Yes	36 (55%)	140 (59%)	0.9 (0.5–1.6)
Grades (school performance)	Below average	12 (18%)	42 (18%)	1.0 (0.4–2.1)
	Average	28 (43%)	107 (45%)	0.9 (0.5–1.7)
	Above average	25 (39%)	88 (37%)	Baseline
**Quality of life**				**p-value**
Total score		48.4 (42.2)	49.4 (43.6)	0.33
Physical health		81.4 (19.4)	85.3 (17.8)	0.09
Emotional functioning		82.8 (15.6)	86.8 (13.0)	0.02
Social functioning		91.1 (17.5)	92.8 (12.8)	0.32
School functioning		72.3 (19.1)	77.2 (16.2)	0.04

Among children above 5 years, those with hearing loss tended to be less likely to be attending school. However, if the child was attending school, hearing loss did not seem to affect whether they were in the correct class at school for their age or their school performance. For those children not attending school, lack of money was the main factor (35%), while lack of transport (23%) and child too sick (19%) were other major factors; the remaining did not identify the reason. Children with hearing loss reported poorer emotional and school functioning, but not poorer overall quality of life, physical health or social functioning.

## Discussion

The overall prevalence of hearing loss in any ear among children with HIV in this study was 24%, and was largely conductive in nature. Overall, 5.5% of all children tested required a hearing aid, almost one in four of those with hearing loss. Few of the children with hearing loss had previously undergone audiometry or had been referred for relevant services. Factors significantly associated with hearing loss included WHO Stage 3 or 4 at enrollment, perceived hearing loss by caregiver, documentation of hearing or ear problems in the medical record, history of frequent ear infections or ear drainage, and positive screens for reported signs of hearing impairment. Children with HIV and hearing loss were more likely to report other disabilities, less likely to attend school, and had poorer emotional and school functioning.

Most previous studies have reported the prevalence of hearing loss among children living with HIV to be 20–30% [8–13,16], and so our findings are within this range. Other studies have reported a higher prevalence of hearing loss among children living with HIV than our estimates [14,17], potentially because the participants in our study were generally healthier, for instance with a low proportion of patients with WHO Stage III/IV. Furthermore, the BCOE is a tertiary health center with more resources than most health centers in Malawi, including equipment for testing hearing (e.g. otoscopes), and a consistent supply of oral and topical antibiotics to treat acute otitis media and chronic suppurative otitis media.

When reviewing clinical factors associated with hearing loss in HIV-infected children, we found that hearing loss was more likely in those with more severe illness at enrollment (WHO Stage 3 or 4), similar to other studies where hearing loss was more common in children with history of AIDS-defining illnesses [8]. Our findings that current disease status (ie: WHO Stage) and nadir CD4 were not found to be statistically significant supports previous findings [8].

Predominant types of hearing loss in HIV-infected children have varied across studies, with several studies reporting the majority with conductive hearing loss [10,16], as in this study, while others studies reported higher prevalence of sensorineural hearing loss [8,9,15] or central auditory disorders [14]. The high prevalence of conductive hearing loss in this and other studies was likely due to high rates of ear infections, as we saw high rates of middle ear abnormalities, such as perforated tympanic membranes.

This study shows a large unmet need for ear examination and hearing services among children living with HIV in Malawi. This finding is concerning, especially given that the vast majority of children were already benefiting from ART and therefore in regular contact with health care professionals. Caregivers were unreliable at perceiving hearing loss: only 40% of caregivers of children with hearing loss accurately perceived the hearing loss, while 11% of children with no hearing loss were reported by their caregiver to have difficulties hearing. One possible explanation may be that the definition of hearing loss used in this study (>20 dB HL in either ear), resulting in a lack of recognition of hearing loss in one ear. Possible explanations for over-reporting of hearing loss in children with normal audiologic exams may be due to the fluctuating nature of conductive hearing loss, where previous conductive hearing loss has resolved. Additionally, there is likely to be high prevalence of cognitive and developmental delay [24,25] and central auditory processing disorders [14] among these children, and caregivers may therefore have found it difficult to distinguish between these disorders and hearing loss, resulting in higher caregiver report of hearing loss in children with normal audiologic testing. These findings imply that parents are not sufficiently reliable judges to allow the identification and referral of children with hearing loss for services. Routine screening for all HIV-infected children with OAE would detect a high proportion of the ears with hearing loss, and is now possible at relatively low cost by non-specialists with widely available mobile phone technology. Alternatively, hearing loss screening questions could be developed to detect those at high risk for hearing loss so that they can be referred for ear exam and hearing evaluation (e.g. those with perceived hearing impairment, frequent ear infections, ear drainage, TB, more severe HIV or a low BMI), although this study showed that screening questions alone missed many children with hearing loss, and a better screening tool for hearing loss needs to be developed. Education regarding ear health and hearing loss in HIV-infected children is needed for both clinicians as well as patients and their caregivers.

In our patients, the high prevalence of conductive hearing loss and high frequency of discharging ears and abnormal tympanic membranes suggest recurrent or chronic otitis as a likely cause of hearing loss. This finding implies that preventive services could focus on the early identification and treatment of ear infections in these children, and different models of developing this intervention should be considered. Furthermore, many of the children with hearing loss could benefit from hearing aids, indicating another point for intervention. Studies are needed to develop screening strategies for hearing loss among children with HIV, as well as prevention and treatment services.

Hearing loss related to HIV was demonstrated to impact school attendance and aspects of quality of life, which has not been described previously, and further contributes towards the disadvantages experienced by children with HIV. These impacts may be ameliorated through the provision of hearing aids. Other interventions may also be needed to ensure that children with hearing loss can participate fully in school, such as training of teachers or linking the children with hearing loss to community-based rehabilitation programmes.

Strengths of this study include a large sample size, with comprehensive assessments of the ears and hearing completed for every child. A broad range of data was collected through the electronic medical record, patient and caregiver interviews, and formal audiologic assessment. Limitations of this study include lack of an HIV-negative control group. However, data on children in Africa show that the expected prevalence of hearing loss is far lower than that identified in this study [26]. Another limitation is the lack of consistency in the definition of hearing loss. While the World Health Organization (WHO) recommends 25dB or greater as the criteria for hearing loss [27], studies commissioned through the WHO have used 20dB as the cutoff [26], and 20dB is widely considered among audiologists as the cutoff for normal hearing. Furthermore, several studies of hearing loss in HIV-infected children have even used 15dB as the cutoff [13,16–17]. Therefore, comparison of prevalence among studies can be difficult when different cutoffs are used. The prevalence of hearing loss was lower than expected, so the study was underpowered for some responses, such as CD4<200. There were only 36 children with hearing loss > 20 dBHL in the better ear, so additional analyses of this subset of children would not have been statistically robust. As our patient population attends care at a tertiary health facility, results may not be representative of all HIV-infected children in Malawi; therefore, further testing needs to done in more typical health centers in Malawi. Exposure to ototoxic medications such as quinine and gentamicin was not included in the analysis, as these medications are usually given in the inpatient setting and are thus not regularly recorded in the clinic EMR. Etiologies of hearing loss were unable to be determined due to limitations in diagnostic testing for diseases such as cytomegalovirus (CMV), so history of such illnesses was unable to be included in the study.

## Conclusions

There is an urgent need for improved screening tools, identification and treatment of hearing problems in HIV-infected children, as hearing loss was common in this group and affected school functioning and quality of life. Clear strategies were identified for prevention and treatment, since most hearing loss was conductive in nature, likely due to frequent ear infections, and many children with hearing loss qualified for hearing aids. Screening strategies need to be developed and tested, since caregivers were not reliable at identifying hearing loss, and often mis-identified children with normal hearing as having hearing loss. Children with frequent ear infections, ear drainage, TB, severe HIV disease, or low BMI should receive more frequent ear assessments and hearing evaluations.
